# Cross‐species models of human melanoma

**DOI:** 10.1002/path.4632

**Published:** 2015-10-09

**Authors:** Louise van der Weyden, E Elizabeth Patton, Geoffrey A Wood, Alastair K Foote, Thomas Brenn, Mark J Arends, David J Adams

**Affiliations:** ^1^ Experimental Cancer GeneticsThe Wellcome Trust Sanger InstituteHinxtonCambridgeshireCB10 1SAUK; ^2^ MRC Human Genetics Unit, The MRC Institute of Genetics and Molecular MedicineThe University of Edinburgh, Western General HospitalCrewe RoadEdinburghEH4 2XUUK; ^3^ Department of Pathobiology, Ontario Veterinary CollegeUniversity of Guelph50 Stone Road EGuelphOntarioN1G 2W1Canada; ^4^ Rossdales Equine HospitalCotton End Road, ExningNewmarketSuffolkCB8 7NNUK; ^5^ Pathology DepartmentWestern General HospitalCrewe RoadEdinburghEH4 2XUUK; ^6^ Centre for Comparative PathologyUniversity of Edinburgh, Western General HospitalCrewe Road SouthEdinburghEH4 2XRUK

**Keywords:** melanoma, cross‐species analysis, genome analysis

## Abstract

Although transformation of melanocytes to melanoma is rare, the rapid growth, systemic spread, as well as the chemoresistance of melanoma present significant challenges for patient care. Here we review animal models of melanoma, including murine, canine, equine, and zebrafish models, and detail the immense contribution these models have made to our knowledge of human melanoma development, and to melanocyte biology. We also highlight the opportunities for cross‐species comparative genomic studies of melanoma to identify the key molecular events that drive this complex disease. © 2015 The Authors. *The Journal of Pathology* published by John Wiley & Sons Ltd on behalf of Pathological Society of Great Britain and Ireland.

## Introduction

Melanocytes produce melanin that protects skin from the effects of ultraviolet light, and also reside as part of mucosal tissues at sites such as the lower bowel, anus, vulva, mouth, and upper aero‐digestive tract. Melanocytes are also found in the uvea/iris of the eye and in the inner ear.

### Genetic predisposition to melanoma in humans

**Figure 1:**
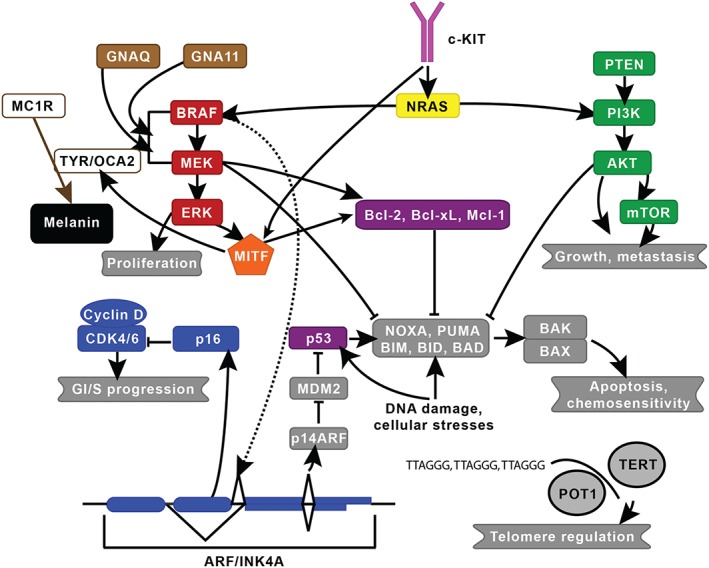
Established melanoma pathways. The two major signalling pathways implicated in melanoma are the mitogen‐activated protein kinase and the phosphatidylinositol‐4,5‐bisphosphate 3‐kinase pathways, which are in red and green, respectively. Key genes include c‐KIT (pink), CDK (blue), GNAQ/GNA11 (brown), MITF (orange), NRAS (yellow), and P53/BCL (purple). MC1R, which is involved in skin pigmentation, and TERT and POT1, which are involved in telomere regulation, are also shown. This figure was modified from Vidwans et al 142 under the Creative Commons Attribution License. The lines shown indicate known interactions between pathways or molecules.

Cutaneous melanoma is largely a malignancy of fair‐skinned people with familial and sporadic genetic risk factors. Population‐based genome‐wide association studies (GWAS) have been particularly informative at defining the relevant melanoma risk regions in the sporadic disease, and to date, around 20 genome‐wide significant loci have been identified 1, 2. These include regions surrounding the melanocortin 1 receptor (*MC1R*) and tyrosinase (*TYR*) genes. MC1R is a G‐protein coupled receptor located in the plasma membrane that plays an important role in controlling the microphthalmia‐associated transcription factor (*MITF*) gene (Figure 1). Disruptive mutations of *MC1R* are associated with red hair, freckling, and sun sensitivity due to a failure in the processing of red/yellow pheomelanin to brown/black eumelanin. Importantly, the function of *MC1R* is highly conserved across species and contributes to skin colouration in a range of higher vertebrates and in fish 1, 3. Tyrosinase is the rate‐limiting enzyme in the production of melanin (variants of which include eumelanin and pheomelanin, described above); *TYR* is transcriptionally regulated by MITF binding to its promoter. The oxidase activity of tyrosinase converts dopa to dopaquinone, a precursor of melanin. Mutations in tyrosinase result in a rare disorder called oculocutaneous albinism 4, 5, which is associated with ultraviolet (UV) sensitivity, while common variants associated with blue eyes have been linked to melanoma predisposition by GWAS 2. In addition to the *MC1R* and *TYR* genes, a variant (E318K) in the aforementioned *MITF* gene is associated with increased melanoma risk in sporadic and familial cases, and is an intermediate genetic risk factor 6. Genes linked to naevus density have also been implicated in disease development (such as *PLA2G6* and *IRF4*), with common variants in or near these genes being revealed by GWAS 2, 7. Likewise, melanoma GWAS have identified variants linked to the cyclin‐dependent kinase inhibitor 2A (*CDKN2A*) gene 1, 2. Importantly, loss‐of‐function mutations in *CDKN2A*, and its binding partner, the product of the cyclin‐dependent kinase 4 (*CDK4*) gene, have been identified in highly melanoma‐prone families 8, 9, 10, firmly linking disruption of cell cycle control and melanoma risk. In addition to the abovementioned genes, recent work has implicated components of the telomere regulation machinery as playing important roles in melanomagenesis. First, the telomerase reverse transcriptase gene (*TERT*) was identified in GWAS studies, and by the analysis of a familial melanoma pedigree, a −57 bp mutation creating an ETS transcription factor binding site that activates the *TERT* promoter was identified 11. More recently, melanoma family studies have identified the protection of telomeres 1 gene (*POT1*), with mutations in the DNA binding domain of POT1 dramatically increasing telomere length and promoting telomere fragility 12, 13. POT1 is a component of the shelterin complex, which is a key regulator of telomere end‐processing. Other components of this complex have been shown to be truncated in melanoma‐prone families including *ACD* and *TERF2IP*, but the exact consequences of these mutations on telomere function are yet to be defined 14.

While these genetic susceptibility studies have defined the landscape of predisposition to cutaneous melanoma, less is known about the germline genetic contribution to other forms of melanoma. At present, we know that rare loss‐of‐function variants in the *BAP1* gene, encoding the BRCA1‐associated protein‐1 (ubiquitin carboxy‐terminal hydrolase), a deubiquitinating enzyme, predispose to ocular melanoma, with some patients also developing cutaneous melanomas and tumours of other sites 15, 16, yet these mutations account for less than 10% of uveal melanoma families. Little is known about the germline genetics of acral and mucosal melanoma, due largely to the rarity of these forms of the disease, and no association with pigmentation genes has been observed 17. Some reports have suggested that patients with Werner syndrome, a DNA repair and ageing syndrome, have an increased incidence of these cancers 17.

### Genetic predisposition to melanoma in animals

With the exception of mutations in *MC1R*, few studies have addressed the genetics of predisposition to melanoma development in animal models. The studies that have been performed have again revealing an important role for genes that influence pigmentation in the cutaneous disease. In horses, for example, a 4.6 kb intronic mutation in the *STX17* (syntaxin‐17) gene was found to be associated with a vitiligo‐like depigmentation phenotype and susceptibility to melanoma 18. Cutaneous melanoma also occurs in dogs, with some reports of differences in breed susceptibility suggesting a genetic basis for melanoma risk 19. Whether melanoma susceptibility in dogs and horses, or indeed in mice and fish, is mediated via the same genes and pathways as those in humans is not known. Thus, there is a significant opportunity to use genetic studies in animal models to define new genes and loci that influence melanoma risk, and to use these data to guide genetic studies in humans.

### Somatic mutations in human melanoma

Most of what has been learnt about the somatic genetics of cutaneous melanoma in humans has been revealed in the last 15 years. The family studies that identified germline mutations in *CDKN2A* led to the identification of somatic mutation in this gene, and also deletions of the entire gene locus 9, 20. Likewise, studies that identified the phosphatase and tensin homolog (*PTEN*) gene as being mutated in glioma led to the identification of mutations and deletions of this locus in melanoma 21. Studies in mouse melanoma models have contributed significantly to our understanding of melanoma and have established a key role for the mitogen‐activated protein kinase (MAPK) pathway in melanoma development. In early studies, a *HRAS^G12V^* transgene was expressed in melanocytes, resulting in highly penetrant melanoma formation 22. Several years later, activating mutations of the neuroblastoma RAS viral (v‐ras) oncogene homolog (*NRAS*) gene were identified in human melanomas at a frequency of 20% 23, 24, 25, and in 2002, amplicon sequencing studies of melanoma cell lines resulted in the identification of activating *BRAF* mutations, most converting a valine at position 600 to a glutamic acid (BRAF^V600E^), generating a constitutively active kinase in around 50% of cases 26. The discovery of this somatic mutation resulting in constitutive activation of the MAPK pathway has revolutionized the field of melanoma genetics and has recently facilitated a revolution in new therapeutics, with clinically approved agents targeting mutant BRAF, and MEK and ERK now in use 27. More recently, next‐generation sequencing of melanomas and matched germline DNA has identified as many as 20 genes as being statistically significantly mutated in human melanoma 28, 29, 30. Many of these genes are components of the MAPK and phosphoinositide 3‐kinase (PI3K) pathways, or cell cycle regulatory genes such as the protein phosphatase 6, catalytic subunit (*PPP6C*), in addition to regulators of the chromatin landscape (*ARID2*, *ARID1A*, and *ARID1B*) 31, 32, 33. While these studies have defined the complexity of melanoma, they have also challenged the one‐size‐fits‐all approach to disease management. Less is known about the genetics of the non‐cutaneous forms of melanoma but acral, uveal, and mucosal melanomas tend to harbour mutations in the guanine nucleotide‐binding protein G(q) subunit alpha (*GNAQ*), the guanine nucleotide binding protein (G protein), alpha 11 (Gq class) (*GNA11*), and v‐kit Hardy‐Zuckerman 4 feline sarcoma viral oncogene homolog (*c‐KIT*) genes 17. Figure 1 provides an overview of the established melanoma pathways.

### Next‐generation sequencing of human melanomas brings ‘the end of the beginning’ for melanoma gene discovery

To date, around 500 human melanoma germline/tumour pairs have been sequenced, with these tumours being largely of cutaneous origin and a limited number of acral and mucosal origin 28, 29, 30, 34, 35. Likewise, a limited number of uveal melanomas have been sequenced 28, 30, although The Cancer Genome Atlas (TCGA) is currently collecting tumours for sequencing and analysis. So what have these sequencing studies actually taught us? First, the sequence has given us a clearer view of the frequency of mutations in known driver genes. Prior to these studies, we knew, for example, that *BRAF* and *NRAS* mutations occurred but tumour sequencing studies have helped us to resolve their absolute prevalence, particularly at positions outside of the canonical BRAF^V600^ and NRAS^Q61^ residues. Secondly, sequencing has identified new genes. For example, hotspot mutations in *PPP6C* and *RAC1* have been identified, and represent potential sites for therapeutic targeting. We knew previously that RAC GTPase activity could contribute to melanoma development 36, but we had no clear way of grappling with it mechanistically, or for exploiting this knowledge in the clinic. The sequence of human melanomas has also taught us something of the constellations of mutations that occur within individual tumours. We know now, for example, that the RAC1 P29S hotspot mutation is mutually exclusive from mutations in other components of the Rho family, a result that would be predicted, but is clarifying nonetheless 30. We have also learnt that there is a third subtype of cutaneous disease called *NRAS/BRAF* wild‐type melanoma, which is characterized by a high C > T mutation burden, amplifications and mutation of *c‐KIT*, and alterations of *NF1* 28, 30. More recently, a fourth subclass called *NRAS/BRAF/NF1* wild type has been proposed 29. While these studies have been broadly informative, melanomas rarely contain the same complement of mutations, so how specific mutations co‐operate to promote tumour formation remains unanswered. Sequencing of human tumours has also informed us that the major mutagen in cutaneous melanoma is UV light which drives a C > T mutation pattern. It has also revealed the potential role of other processes such as oxidative stress 30. Despite the apparent clarity that sequencing has provided, there is still much to learn, and integrating the sequence data with functional studies, and studies in model systems will be key.

## Using the mouse to model melanoma

There are multiple ways to model melanoma in the mouse, to allow the identification of key oncogenes/tumour suppressor genes involved in melanoma initiation, progression, and metastasis, as well as for the preclinical testing of therapeutics. Here we will outline the use of patient‐derived xenografts, genetically engineered mice, and melanoma cell lines.

### Melanoma cell lines

Established human and mouse melanoma cell lines continue to be workhorses for mechanistic studies, as much can be gained from their analysis. The B16 mouse melanoma model was created in the 1970s from a melanoma that developed spontaneously in a C57BL/6 mouse and was then passaged *in vivo* through ten rounds of tail vein injection and collection of subsequent pulmonary metastases to create the B16‐F10 line 37. This cell line has been used in a plethora of tumour immunology and metastasis studies 37, 38.

*In vitro* work has its limitations (such as a lack of extracellular matrix and 3D growth), and thus, the use of human cell lines *in vivo* is frequently used to model melanoma, usually by subcutaneous injection/engraftment into immunodeficient mice [typically non‐obese diabetic/severe combined immunodeficient (NOD/SCID) mice] that do not produce lymphocytes or NK cells. These cell line xenograft models allow melanoma cells to directly establish interactions with the stroma, the lymphatic system, and blood vessels. Cell line xenografts have been widely used in determining drug responses 39; however, cell lines are undeniably altered during adaptation to *in vitro* conditions and long‐term culture, limiting their usefulness in certain aspects of modelling human melanoma. In particular, their ability to predict clinical drug responses has been widely questioned and critiqued 40.

### Patient‐derived xenograft (PDX) models

Patient‐derived xenograft (PDX) or ‘tumourgraft’ samples are collected under ethical approval as fresh biopsy tissue or fine needle aspirates and within hours implanted subcutaneously into immunodeficient mice 41. A high degree of similarity, at the level of expression and DNA sequence, has been demonstrated between PDXs and donor tumours, with human melanoma PDXs found to be predictive of metastasis 42. Excitingly, the utility of PDXs for informing patient care was recently demonstrated, with PDXs found to be predictive of patient drug response 43.

PDX models, however, do have several important limitations – in particular, the absence of a fully functional immune system – although it is possible to generate partially ‘humanized mice’ using patient‐derived CD34+ haematopoietic stem cells capturing some elements of the human immune system 44.

### Genetically engineered mice (GEM) models

**Figure 2:**
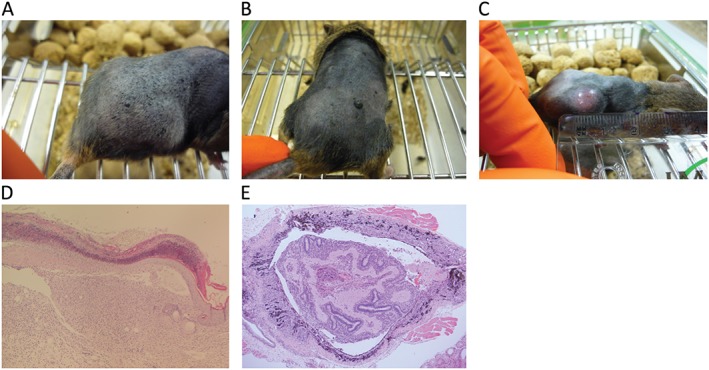
Naevi and melanomas driven by oncogenic forms of Braf and NRAS. (A, B) Naevi developing in adult Braf^V618E^ mice 48. Naevi generally became visible 6–8 weeks after the induction of Braf^V618E^ expression. (C, D) Melanoma from the same Braf^V618E^ model. D shows an invasive malignant melanoma with evidence of infiltration and destruction of the overlying surface epithelium and invasion into the subcutaneous adipose tissue. The average latency to melanoma formation was 426 days in this model. (E) H&E‐stained section of an ocular melanoma, with melanoma cell infiltration of the lens and the subretinal tissues, that developed in a 13‐week‐old Tyr::NRAS^Q61K^ mouse 135. Original magnification × 50.

Although mice rarely develop melanoma spontaneously, they can do so when genetically engineered to carry defined mutations that mimic the genetic lesions (or their consequences) found in human melanomas. These engineered mutations can result in activation of oncogenes (such as mutant *Braf^V600E^* or *Nras^Q61R^*) and/or inactivation of key tumour suppressor genes (such as *Cdkn2a* or *Pten*). Examples of GEM models are listed in Table 1 and shown in Figure 2. In mice, genetic modification of the germline melanoma susceptibility gene *Cdkn2a* (*p16Ink4a* null mice) does not result in melanoma, with these mice typically developing soft‐tissue sarcomas and lymphomas 45. One way around this is to use of compound GEM models. For example, mice carrying melanocyte‐specific (tyrosinase promoter‐controlled) expression of activated *HRAS^G12V^* on an *Ink4a*‐deficient background develop spontaneous cutaneous melanomas after a short latency and with a high penetrance 22. Interestingly, in addition to cutaneous melanomas, these mice also developed ocular melanomas, as has been reported for mice carrying melanocyte‐specific expression of activated *NRAS^Q61K^* on an *Ink4a*‐deficient background (Figure 2) 46. Mutational activation of BRAF in mice carrying conditional melanocyte‐specific expression of *Braf^V600E^* (or the mouse equivalent, *Braf^V618E^*) has also been used to model melanoma development, tumour progression, and drug resistance 47, 48, 49.

**Table 1:** Examples of genetically engineered mouse (GEM) models of melanoma

**GEM name**	**Genes involved**	**Phenotype**	**Reference**
*MT/ret*	Transgenic mice with the *RET* proto‐oncogene fused to the mouse metallothionein promoter‐enhancer	Mice develop hyperpigmented skin due to aberrant melanogenesis and melanocytic tumours develop but do not metastasize The transgenic line ‘304/B6’ (which has been back‐crossed to C57BL/6 for ten generations) spontaneously develops systemic skin melanosis, benign melanocytic tumours, and melanoma that undergoes metastasis to distant organs On a background of *Ednrb* heterozygosity, these mice show late‐onset melanoma development with a high percentage of metastasis, and poor prognosis after tumour development	Iwamoto *et al* 136 Kato *et al* 137 Kumasaka *et al* 138
*HGF/SF*	Transgenic mice with the metallothionein promoter driving overexpression of hepatocyte growth factor/scatter factor (HGF/SF)	The skin of these mice has melanocytes in the dermis, epidermis, and dermal–epidermal junction, and thus this model is more akin to human skin Aged *HGF/SF*‐transgenic mice develop sporadic melanoma with metastasis Cutaneous melanomas arise in UV‐irradiated *HGF/SF*‐transgenic mice in distinct stages that resemble human disease, including grossly identifiable premalignant lesions, intermediate radial and vertical growth stages of heterogeneous histopathologies, and late metastatic spread to a variety of distant organs	Takayama *et al* 139 Noonan *et al* 50, 140
*p16^Ink4a−/−^*	A *p16^Ink4a^*‐specific knockout mouse that retains normal *p19^Arf^* function	Although these mice develop melanomas, they more preferentially develop soft‐tissue sarcoma and/or splenic lymphoma	Sharpless *et al* 45
*Tyr‐HRAS*	Transgenic mice with mouse tyrosinase gene promoter driving overexpression of an oncogenic form of *HRAS* (HRAS^G12V^)	These mice spontaneously developed cutaneous and ocular tumours that are locally invasive and do not undergo metastasis The incidence and latency of melanoma development are accelerated on an *Ink4a*‐deficient background	Chin *et al* 22
*Tyr::NRAS^Q61K^*	Transgenic mice with mouse tyrosinase gene promoter driving overexpression of a dominant‐active human *NRAS* (NRAS^Q61K^)	Mice showed hyperpigmented skin and develop cutaneous metastasizing melanoma On an *Ink4a*‐deficient background, > 90% of the mice developed melanomas that at 6 months micro‐invade the epidermis and disseminate to lymph nodes, lung, and liver	Ackermann *et al* 135
*Hgf‐Cdk4^R24C^*	Overexpression of the hepatocyte growth factor (HGF) and an oncogenic mutation in cyclin‐dependent kinase 4 (CDK4)^R24C^	These mice rapidly develop multiple invasive melanomas in the skin following neonatal or adult carcinogen treatment (UV and/or DMBA), which spontaneously metastasize to lymph nodes and lungs Primary DMBA‐induced melanomas have been used to derive cell lines that when subcutaneously administered to C57BL/6 immunocompetent mice, spontaneously develop lung metastases	Tormo *et al* 141 Gaffal *et al* 51 Bald *et al* 52
*LSL‐Braf^V600E^*	Conditional expression of Braf^V600E^ from the endogenous *Braf* locus	When crossed with *Tyr::CreER* mice and tamoxifen was rubbed on their skin, these mice showed skin hyperpigmentation and the appearance of naevi harbouring senescent melanocytes, with ∼70% developing melanomas On a *p16^INK4a^* null background, these mice developed melanoma with increased penetrance and decreased latency	Dhomen *et al* 47
*Braf* ^CA^	Conditional expression of Braf^V600E^ from the endogenous *Braf* gene	When crossed with *Tyr::CreER* mice and tamoxifen is rubbed on their skin, these mice develop benign melanocytic hyperplasias that fail to progress to melanoma over 15–20 months On a *Pten* null background, these mice developed melanoma with 100% penetrance, short latency, and metastases (lymph nodes and lungs) Melanoma development was prevented by inhibitors of mTorc1 (rapamycin) or MEK1/2 (PD325901) but only whilst the drug was being administered (cessation of administration led to melanoma development). Combined rapamycin and PD325901 treatment led to shrinkage of established melanomas	Dankort *et al* 49
*LSL‐Braf^V618E^*	Conditional expression of Braf^V618E^ from the endogenous murine *Braf* gene (Braf^V618E^ is analogous to BRAF^V600E^ in humans).	When crossed with *Tyr::CreER* mice and tamoxifen was rubbed on their skin, these mice showed skin hyperpigmentation and naevi with ∼80% developing melanoma *Sleeping Beauty* insertional mutagenesis in this model accelerated melanoma latency and penetrance. Treatment with the BRAF inhibitor PLX4720 resulted in tumour regression followed by relapse, and analysis of transposon insertion sites in these melanomas identified putative mediators of resistance	Perna *et al* 48

*LSL* = *lox‐STOP‐lox*.

Given the importance of UV light as a key mutagen in the initiation of melanoma, GEM models of UV‐induced melanoma have been developed, such as *HGF/SF* mice, which were used to show that a single dose of burning UV radiation to neonates, but not adults, is necessary and sufficient to induce melanomas with high penetrance, thus providing experimental support for epidemiological evidence that suggests that childhood sunburn poses a significant risk for developing melanoma 50. More recently, neonatal UVB exposure was shown to accelerate melanoma growth and enhance distant metastases in *Hgf‐Cdk4^R24C^* mice 51, and pulmonary metastasis of melanomas in adult *Hgf‐Cdk4^R24C^* mice was found to occur after treatment with the mutagen DMBA and repeated UVB exposure 52. The *Hgf‐Cdk4^R24C^* model has also been used to generate metastatic melanoma by introducing *p16‐null* and *Nme23*‐null alleles 53, 54.

GEM models have the advantage over other systems of autochthonous tumour development in an environment where the tumour cells interact reciprocally with the immune system and other components of the microenvironment. Disadvantages of GEM models include their expense and the fact that tumours often arise after a long latency (9–12 months) and generally do not carry the mutagenic load found in human tumours. Regardless of these factors, these models have made a fundamental contribution to our understanding of melanoma development.

## Using the dog to model melanoma

### Dogs as spontaneous models of melanoma

Malignant melanoma is a relatively common cancer in domestic dogs and represents a unique model of human melanoma that is highly heterogeneous and arises and metastasizes spontaneously in an immunocompetent animal. There is potential to relate the molecular character of individual tumours to clinical outcome, as pet dogs receive therapy ranging from surgery, radiation, and cytotoxic chemotherapy through to molecularly targeted therapy and immunotherapy. Here we will review the utility of canine melanoma as a comparative model and as a preclinical model of human melanoma.

### Incidence, anatomic location, and clinical progression of melanoma in dogs

**Figure 3:**
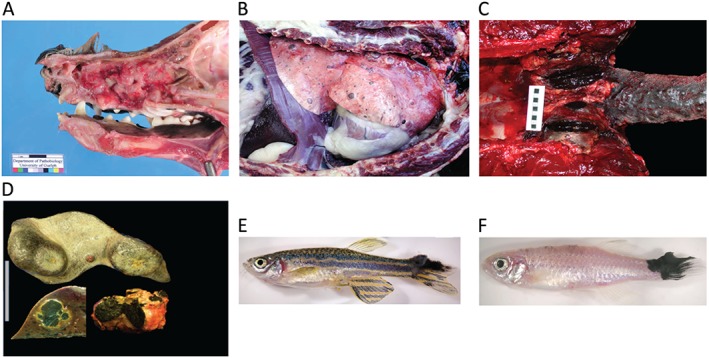
Canine, equine, and zebrafish melanoma. (A) A canine melanoma developing in the nasal cavity and (B) spreading to the viscera, particularly the liver. (C) An equine melanoma showing multinodular dermal lesions around the tail base and masses expanding into the pelvic canal and regional nodes. (D) An equine spleen with multiple malignant melanomas, and liver and lymph node from the same case. (E) Melanomas arising in a BRAF^V600E^; mitf zebrafish and (F) in a BRAF^V600E^; p53 zebrafish. The photographs in A and B were kindly provided by Jeff Caswell, Department of Pathobiology, University of Guelph, Guelph, Ontario, Canada N1G2W1.

Many domestic animals develop spontaneous melanocytic neoplasms, including dogs, cats, horses, and pigs. Malignant melanoma is more common in the dog compared with other species and the majority of cases arise in the oral cavity (mucosal), with haired skin (cutaneous), nailbed epithelium and footpad (subungual and acral), and ocular (uveal) locations being less common 55 (Figure 3). Canine oral melanoma is highly aggressive with frequent metastases, especially to local lymph nodes and lungs. In contrast to human cutaneous melanomas, dog cutaneous melanomas are most often benign 55. Some dog breeds are over‐represented in oral melanoma studies and may be predisposed to developing the disease. In a study of 2350 dogs with melanocytic tumours, poodles, Beauce shepherds, rottweilers, schnauzers, Scottish terriers, and Labrador retrievers had a higher percentage of these tumours than other breeds 56. In general, this study also found that black‐coated breeds were over‐represented and that pale or white‐coated breeds were under‐represented in terms of developing melanocytic tumours. Conventional treatment for oral melanoma in dogs involves surgical resection and/or radiation of the primary tumour to control local disease 57, while treatment of metastatic disease is much less successful. Most metastases are resistant to chemotherapy and a variety of immunotherapeutic approaches have been attempted 58. A commercially produced melanoma vaccine (ONCEPT, Sanofi) is available and has shown efficacy in canine melanoma compared with historical controls 59, but there is controversy as to the level of effect when there has not been a randomized trial 60, and some studies have failed to show an effect on clinical outcome 61. Other immunomodulatory approaches have been applied experimentally in small groups of dogs and show some potential 62, 63. As described in more detail below, there are a number of receptor tyrosine kinase genes that are mutated in canine melanomas and tyrosine kinase inhibitor drugs are already commercially available and used clinically in canine cancer patients 64, but to date no trials have been published using these compounds in canine melanoma.

### Genetics of canine melanoma

The complete canine genome sequence was first released in 2005 65, revealing a significant shared ancestral sequence in common with humans. Indeed, canine DNA and protein sequences are more similar to humans than are those of mice 65. Due to this similarity, molecular tools for studying canine diseases are quite advanced, especially since a large proportion of antibodies raised against human antigens work equally well against canine proteins. Canine oral melanoma does not have UV radiation as a risk factor, so it is not surprising that the spectrum of mutations differs from human cutaneous melanoma. The BRAF^V600E^ mutation is found in about 6% of canine oral melanomas 66, as are non‐canonical *BRAF* mutations. *NRAS* mutations have also been found at the same location as those in human melanoma (the residue corresponding to Q61), and loss‐of‐function mutations in *PTEN* have been reported 56. Also similar to humans, loss of *PTEN* expression, and *c‐KIT* mutation, and/or overexpression of *c‐KIT* are common in the canine disease. Importantly, comparative copy number studies have been performed between dog and human melanomas of mucosal and acral origin, suggesting that in concordance with what is known for human melanomas, canine melanomas of the oral mucosa and cutaneous epithelium are discrete and initiated by different molecular pathways 67.

### Canine melanoma as a preclinical model

The overall success rate of translating cancer therapies from murine preclinical models to treatments with clinical utility in humans is estimated to be around 5% 68. Although the increasingly sophisticated mouse *in vivo* modelling systems described above are more likely to capture the complexity of human cancer than the less refined systems used in the past, a complementary approach is to include dogs with spontaneous cancers 69. Until recently, humans could be considered a preclinical model for dogs; the majority of drugs used in veterinary medicine are derived from drugs initially designed and tested for efficacy in humans. There is an organized network for conducting clinical trials in dogs with cancer across the United States and Canada organized through the National Cancer Institute, called the Comparative Oncology Trials Consortium 70, 71. This allows for multi‐centre clinical trials with defined inclusion and exclusion criteria, much like human clinical trials 71. From the perspective of the veterinary patients and their owners, the access to investigational therapies is a clinical trial; from the perspective of human patients, these can be thought of as preclinical trials. This has led to the concept of co‐clinical trials, where both human and canine patients with the same tumour type, or mutation spectrum, receive the same drugs 72. In addition to mirroring the heterogeneity and complexity of spontaneously arising cancer, dog trials have other practical advantages; the contracted disease timeline allows earlier assessment of effects on disease progression and overall survival, since the lifespan of dogs is far shorter than that of humans and canine cancers progress more quickly in general. Furthermore, new drugs are commonly tested for toxicity in laboratory beagles, so the initial safety and sometimes the pharmacodynamics and pharmacokinetics are already known for dogs.

A recent, excellent study by Simpson *et al* 73 explored the utility of canine melanoma as a model of the human disease, and readers are referred there for a more in‐depth review. The consensus of that group was that there are substantial clinical and histopathological similarities between mucosal melanomas in the two species. The Simpson study leveraged the Canine Comparative Oncology and Genomics Consortium (http://www.CCOGC.net), which contains a large collection of canine tumours including matched dog melanoma/normal pairs. To date, there have been relatively few large clinical trials in canine melanoma. The development of the canine melanoma vaccine introduced above involved only 58 patients 59. As in humans, the majority of melanoma therapies tried in dogs have failed, which although discouraging, might be considered evidence for the utility of canine melanoma as a model. As canine melanoma is a heterogeneous cancer, has developed in the context of an intact immune system, and occurs in a genetically heterogeneous population of animals, only the most robust investigational drugs will be able to show efficacy in a clinical trial. Thus, although the majority of melanomas forming in dogs are of mucosal origin, and thus rarer in frequency than common melanomas in humans which are cutaneous, there are significant opportunities in studying dog melanomas alongside those of human and other species.

## Using the horse to model melanoma

### Incidence, anatomic location, and clinical progression of melanocytic tumours in horses

Melanocytic tumours are common tumours of horses, representing approximately 4–8% of all tumours 74 and up to 19% of cutaneous tumours 75, 76.

As in other species, the terminology and classification of melanocytic tumours in horses has been inconsistent over time and has led to confusion between clinicians and pathologists 77. Four clinical syndromes are currently recognized in horses: melanocytic naevi (sometimes referred to as melanocytoma); dermal melanomas; dermal melanomatosis; and anaplastic malignant melanoma 78. Some of the melanocytic naevi resemble human naevi 79, and these occur in both grey and non‐grey horses, usually on the legs, body or neck rather than the perineal region. Equine dermal melanomas and dermal melanomatosis are histopathologically similar, distinguished by their clinical presentation; the former tend to be solitary discrete tumours, whereas dermal melanomatosis presents as multifocal dermal lesions, often coalescing and usually occurring in typical locations (most commonly the genital or tail base/perineal region, and less commonly periocular and perioral). Dermal melanomatosis is a disease of grey and white horses, and beyond the age of 15 years, at least 80% of grey horses will have melanomas at some location 78, 80. While they usually have a benign initial presentation, they often develop multi‐centrically and are often associated with blood vessels [for example, in the wall of the guttural pouches (paired air‐filled chambers formed from outpouching of the Eustacian tube), around the parotid salivary glands and lymph nodes, paralumbar, peri‐aortic and neck/carotid region]. In addition, many will progress to true malignant forms with lymphatic and visceral metastases 81. Malignant forms occur in both grey and non‐grey horses, although the risk of malignant transformation may be greater in non‐grey horses 75. At least in grey horses, histopathological features do not reliably predict malignant behaviour 82, although application of new biomarkers, such as RACK1, may show promise 83. Ocular 84 and mucosal melanomas 85, 86 are far less common in horses than in other domestic species.

### Equine melanoma as a comparative model

As in canine melanomas, equine melanomas are not thought to be associated with exposure to UV light. Development of the grey hair coat colour in horses with age is an autosomal dominant trait associated with a high incidence of melanoma, and also vitiligo‐like depigmentation 18. The causative mutation for this phenotype is a 4.6‐kb intronic duplication in the *STX17* (syntaxin 17) gene, which constitutes a *cis*‐acting regulatory mutation. Both *STX17* and the neighbouring *NR4A3* gene are overexpressed in melanomas from grey horses. It is known that the duplication in *STX17* is strongly associated with constitutive activation of the ERK pathway in melanocytic cells from grey horses, highlighting the universal importance of the MAPK/ERK pathway in melanomagenesis 87. Further, experimental models using reporter constructs in transgenic zebrafish have demonstrated that the duplicated *STX17* sequence acts as a strong enhancer in neural crest cells and has subsequent melanophore‐specific activity during embryonic development, consistent with the phenotypic manifestation of the mutation in horses 88. This study went on to demonstrate that one region of the construct up‐regulated the reporter gene expression in a melanocyte‐specific manner and contained two microphthalmia‐associated transcription factor (MITF) binding sites, which are good candidates for mediating the melanocyte‐specific activity of the duplication.

As in other species, tumour subtype and breed/individual variation (germline genetics) are likely to influence the phenotype of the melanomas formed 89. Indeed, grey horses that possess a loss‐of‐function mutation in the *ASIP* (agouti signalling protein) gene have a higher incidence of melanoma, implicating melanocortin‐1 receptor signalling in melanoma development in these animals 18.

In terms of biological behaviour, grey horse melanomas usually have an extended period of benign growth, prior to malignant transformation and metastasis, in contrast to most human melanomas, which metastasize early*. In vitro* cell lines of primary and metastatic horse melanomas revealed expression of p53, while expression of the tumour suppressors p16 and PTEN was absent from the metastatic line 90, potentially implicating the latter pathways in disease progression.

In terms of histopathology, animal‐type melanoma in humans represents a rare distinct melanoma subtype, characterized by proliferation of heavily pigmented epithelioid and spindled melanocytes, that resembles the heavily pigmented melanomas seen in grey horses 91, 92. In humans, the disease has a young age of onset (median 35 years old) and is considered to be more indolent than conventional melanoma; it has a tendency for regional lymphatic metastasis but infrequently progresses to disseminated metastatic disease and death. Direct comparison of the genetic and molecular alterations in human and equine melanomas will provide fascinating insights into the mechanisms of melanomagenesis 93.

## Using zebrafish to model melanoma

### The translational impact of zebrafish models of melanoma

Modelling melanoma in zebrafish provides important opportunities for *in vivo* imaging, chemical screens, and genetics. Zebrafish cancers, including melanoma, share many histopathological features with human cancers, and molecular signatures closely align with those of human cancer. Here we outline the use of genetically engineered zebrafish and xenograft models, and discuss how zebrafish have become instrumental for chemical screens for drug leads and repurposing for melanoma.

### Genetically engineered zebrafish (GEZ) models

The zebrafish genome shares over 70% similarity with the human genome, and over 80% of human disease genes – including oncogenes and tumour suppressors – have orthologs in zebrafish 94. Zebrafish cancer models have primarily depended on transgenic expression of oncogenes and *N*‐ethyl‐*N*‐nitrosourea (ENU)‐induced genetic mutations in tumour suppressor genes. However, the advent of genome editing with the clustered regularly interspaced short palindromic repeats (CRISPR) system now enables precise and tissue‐specific genetic editing that will enable more refined genetic modelling of human melanoma 95, 96, 97, 98, 99.

In the first zebrafish melanoma model, human BRAF^V600E^ protein expressed from the melanocyte *mitfa* promoter led to the generation of naevi, and a mutation in *p53* (*p53^−/−^*) was required for progression to melanoma 100. This was the first animal model of the BRAF^V600E^ mutation and was consistent with genetics in human patients whereby expression of BRAF^V600E^ is sufficient to drive naevi, but requires additional mutations for progression of melanoma from naevi 26. Building on the *BRAF^V600E^;p53^−/−^* model, Zon and colleagues generated a modified zebrafish whereby the *BRAF^V600E^* transgene was co‐expressed with one of 17 candidate genes from a recurrently amplified region in human melanoma on chromosome 1q21 101. Screening for genes that promoted the rapid onset of melanoma, they discovered that overexpression of the histone methyltransferase SETDB1 can accelerate the onset and invasion of melanoma. High expression levels of SETDB1 are common in human melanoma and indicate that changes in chromatin factors may be critical in melanoma progression through changes in gene regulation, such as the *hox* genes 101.

An important feature of zebrafish melanoma is the ability to study melanocyte development genes and how the lineage can become misregulated in melanoma 102. The master melanocyte transcription factor MITF is a melanoma oncogene and has been implicated in melanoma drug resistance, but until recently it had not been modelled in an animal. A unique temperature‐sensitive *mitf* mutation in zebrafish (*mitfa^vc7^*) has recently been used to study MITF activity in the control of melanocyte proliferation and differentiation in embryogenesis, and as a cancer gene in the development and survival of melanoma 103, 104, 105.

RAS mutations have also been modelled in zebrafish. Expression of HRAS^G12V^ (HRAS^12V^) protein in *kit‐*expressing melanocyte progenitors is sufficient to drive rapid expansion of melanocyte numbers in the larval form and melanoma in the adult, and this is dependent on PI3K signalling 106. Co‐operation studies have also demonstrated that elevated RAC activity, often associated with melanoma in humans, can accelerate the progression of HRAS^V12^‐driven malignant melanoma 107. While HRAS^V12^ melanoma studies have helped to establish melanoma models important for drug screens and cell biology studies 108, *NRAS* mutations are the common RAS family melanoma mutation, and genetic models in zebrafish indicate that NRAS^Q61K^ mutations in melanocytes require co‐operation with loss of *p53* to promote melanoma 109.

As with mice, limitations of the zebrafish *BRAF^V600E^* models include the lengthy time for spontaneous tumour formation and that genetically engineered animals do not seem to have the diversity and number of mutations found in human melanomas 110. Accelerating tumour formation with HRAS^V12^ mutations enables melanoma to be visualized at the earliest stages in the zebrafish 106, 108. Zebrafish embryos and larvae are transparent, enabling details of cell biology and the lineage to be visualized in living animals. An important example of this is the interactions of the immune system with *HRAS^V12^* oncogene‐expressing melanocytes at the very earliest stages of neoplasia. Immune cells provide trophic support to *HRAS^V12^* oncogene‐expressing melanocytes 111, 112.

### Transplantation models of melanoma in zebrafish

Transplantation assays are fundamental to understanding cancer cell malignancy, migration, and cancer‐initiating cells. Zebrafish provide transplantation studies at three stages: the early embryo, the larvae, and the adult animal 113. Transplantation into the early embryo (prior to gastrulation) has been used to identify important melanoma pathways, such as nodal via the generation of an ectopic developmental axis 114, 115, 116, 117. Transplantation of human cancer cells into the larval stage can lead to melanoma masses within a few days, and enables the study of tumour‐induced vascularization and cancer cell metastatic spread. The availability of lines with fluorescently labelled vasculature, such as fli‐GFP, allows for angiogenesis or lymphoangiogenesis to be visualized in living animals 118, 119. Fluorescently labelled melanoma cells can also be visualized in the process of co‐operative behaviours during invasion in zebrafish embryos 120. An advantage to these early‐stage transplantation studies is the large number of zebrafish that can easily be injected and that can be coupled to live confocal imaging 121. The zebrafish immune system in these early stages primarily consists of innate immune cells, and the adult immune system is not fully functional until 28 days of development 113. In some cases, transplanted melanoma cells have capitalized on neutrophil migration routes to new metastatic niches 122.

Adult transplantation studies in zebrafish have been important for assessing tumour potential, visualizing cancer homing and metastasis, and in competitive assays for tumourigenicity. Important considerations in adult transplantation studies include the need to suppress the immune system. To get around these issues, immunosuppression can be induced by gamma irradiation prior to transplantation (eg 20–25 Gy), or dexamethasone in larval/juvenile fish, and isogenic strains have recently become available 113. Adult zebrafish are no longer transparent, preventing detailed visualization of engrafted tumours in living animals. Recently, a transparent adult fish, called *casper*, has been generated that enables visualization of transplanted melanoma cells – either by their endogenous black pigmentation or via a fluorescent transgene – at the single cell level 102, 123, 124. Limitations of the adult transplantation studies are that human cancer cells do not engraft due to immunogenicity and that most cells are injected via intraperitoneal injection rather than orthotopically 113.

### Small molecule and drug screening in zebrafish

A unique feature of the zebrafish system is the ability to treat the whole organism with drug treatments by administering chemical compounds to the water 125. This approach can be used to directly test the function of a targetable pathway in transplantation studies, to screen for new drug leads during early embryogenesis, and for testing compounds in adult zebrafish cancer models 126. Examples include small molecule screens on the melanocyte lineage that identified 5‐nitrofuran compounds, which are also effective in human melanoma 127, and the changes caused by *BRAF^V600E^;p53* at the embryonic level that identified leflunomide, which is currently in clinical trials for melanoma 126 (Clinical trials.gov identifier NCT01611675). Overall, phenotypic small molecule screening in zebrafish is proving effective at multiple stages of the drug discovery pipeline including hit identification, target identification, lead optimization, and preclinical animal modelling 128, 129.

Other models of melanoma not discussed here include the Sinclair swine model 130, which shows spontaneous regression; the Libechov minipig model 131; and 3D human to mouse transplant models 132. There are also *Xiphophorus* models 133 and an opossum melanoma model 134.

## Conclusion

### Perspectives and relevance of animal models to melanoma in humans

Animal model studies in a range of species confirm the ‘naevus–melanoma’ pathway as the major sequence of pathological progression to melanocytic malignancy. They also establish naevi as neoplasms that have mutations in oncogenes and tumour suppressor genes, as opposed to the previously held pathological view of naevi as non‐neoplastic hamartomas. The experimental animal model studies demonstrate that some cancer genes (such as *BRAF*, *NRAS*, *MITF*, *TP53*, *P16*/*CDKN2A*, *BAP1*, *PTEN*, *C‐KIT*, etc) can drive naevus formation and/or progression to melanoma in various combinations, while sequencing studies of human melanomas emphasize the genetic heterogeneity of the disease with potential for reclassification based on the genetic phenotype in the future. Multi‐species comparative pathology and genomics (human, mouse, zebrafish, dog, horse, other) help to identify new melanoma genes for cutaneous melanoma, mucosal melanoma, and less common melanomas at other sites, including the study of rare subtypes of melanoma. These molecular studies also shed light on melanoma progression genes that influence the stage or aggressive behaviour of the melanoma, potentially contributing to an improved molecular and mechanistic understanding of melanoma progression to metastasis in patients and serving as predictors of outcome or potential therapeutic targets. Small animal models (such as mouse or zebrafish) are informative for preclinical drug testing and investigation of mechanisms of drug resistance, as well as providing new insights into the melanoma–immune system interactions, which are of increasing relevance to patient therapy.

## Author contribution statement

LvdW, EEP, GAW, and AF wrote sections of the manuscript on mouse, zebrafish, dog, and horse melanoma, respectively. TB, MJA, and DJA wrote on the genetics and pathology of human melanoma. All authors contributed to revision of the manuscript and the final published paper. All authors contributed equally.

## References

[1] Hill VK , Gartner JJ , Samuels Y , *et al.* The genetics of melanoma: recent advances. Annu Rev Genomics Hum Genet 2013; 14 **:** 257–279. 23875803 10.1146/annurev-genom-091212-153429

[2] Law MH , Bishop DT , Lee JE , *et al.* Genome‐wide meta‐analysis identifies five new susceptibility loci for cutaneous malignant melanoma. Nature Genet 2015; 47 **:** 987–995. 26237428 10.1038/ng.3373 PMC4557485

[3] Schiöth HB , Raudsepp T , Ringholm A , *et al.* Remarkable synteny conservation of melanocortin receptors in chicken, human, and other vertebrates. Genomics 2003; 81: 504–509. 12706108 10.1016/s0888-7543(03)00028-4

[4] Scherer D , Kumar R . Genetics of pigmentation in skin cancer – a review. Mutat Res 2010; 705: 141–153. 20601102 10.1016/j.mrrev.2010.06.002

[5] Shibahara S. Mutations of the tyrosinase gene in oculocutaneous albinism. Pigment Cell Res 1992; 5: 279–283. 1292010 10.1111/j.1600-0749.1992.tb00550.x

[6] Yokoyama S , Woods SL , Boyle GM , *et al.* A novel recurrent mutation in MITF predisposes to familial and sporadic melanoma. Nature 2011; 480: 99–103. 22080950 10.1038/nature10630 PMC3266855

[7] Duffy DL , Iles MM , Glass D , *et al.* IRF4 variants have age‐specific effects on nevus count and predispose to melanoma. Am J Hum Genet 2010; 87: 6–16. 20602913 10.1016/j.ajhg.2010.05.017 PMC2896771

[8] Kamb A , Shattuck‐Eidens D , Eeles R , *et al.* Analysis of the p16 gene (*CDKN2*) as a candidate for the chromosome 9p melanoma susceptibility locus. Nature Genet 1994; 8: 23–26. 7987388 10.1038/ng0994-22

[9] Hussussian CJ , Struewing JP , Goldstein AM , *et al.* Germline p16 mutations in familial melanoma. Nature Genet 1994; 8: 15–21. 7987387 10.1038/ng0994-15

[10] Ranade K , Hussussian CJ , Sikorski RS , *et al.* Mutations associated with familial melanoma impair p16^INK4^ function. Nature Genet 1995; 10: 114–116. 7647780 10.1038/ng0595-114

[11] Horn S , Figl A , Rachakonda PS , *et al.* TERT promoter mutations in familial and sporadic melanoma. Science 2013; 339: 959–961. 23348503 10.1126/science.1230062

[12] Robles‐Espinoza CD , Harland M , Ramsay AJ , *et al.* POT1 loss‐of‐function variants predispose to familial melanoma. Nature Genet 2014; 46: 478–481. 24686849 10.1038/ng.2947 PMC4266105

[13] Shi J , Yang XR , Ballew B , *et al.* Rare missense variants in POT1 predispose to familial cutaneous malignant melanoma. Nat Genet 2014; 46: 482–486. 24686846 10.1038/ng.2941 PMC4056593

[14] Aoude LG , Pritchard AL , Robles‐Espinoza CD , *et al.* Nonsense mutations in the shelterin complex genes *ACD* and *TERF2IP* in familial melanoma. J Natl Cancer Inst 2015; 107 **:** dju408. 10.1093/jnci/dju408 PMC4334787 25505254

[15] Harbour JW , Onken MD , Roberson EDO , *et al.* Frequent mutation of BAP1 in metastasizing uveal melanomas. Science 2010; 330: 1410–1413. 21051595 10.1126/science.1194472 PMC3087380

[16] Battaglia A. The importance of multidisciplinary approach in early detection of BAP1 tumor predisposition syndrome: clinical management and risk assessment. Clin Med Insights Oncol 2014; 8: 37–47. 24855403 10.4137/CMO.S15239 PMC4011723

[17] Bastian BC . The molecular pathology of melanoma: an integrated taxonomy of melanocytic neoplasia. Annu Rev Pathol 2014; 9: 239–271. 24460190 10.1146/annurev-pathol-012513-104658 PMC4831647

[18] Rosengren Pielberg G , Golovko A , Sundström E , *et al.* A *cis*‐acting regulatory mutation causes premature hair graying and susceptibility to melanoma in the horse. Nature Genet 2008; 40: 1004–1009. 18641652 10.1038/ng.185

[19] Dobson JM . Breed‐predispositions to cancer in pedigree dogs. ISRN Vet Sci 2013; 2013: 941275. 23738139 10.1155/2013/941275 PMC3658424

[20] Puig S , Ruiz A , Lázaro C , *et al.* Chromosome 9p deletions in cutaneous malignant melanoma tumors: the minimal deleted region involves markers outside the p16 (CDKN2) gene. Am J Hum Genet 1995; 57: 395–402. 7668266 PMC1801531

[21] Guldberg P , thor Straten P , Birck A , *et al.* Disruption of the *MMAC1/PTEN* gene by deletion or mutation is a frequent event in malignant melanoma. Cancer Res 1997; 57: 3660–3663. 9288767

[22] Chin L , Pomerantz J , Polsky D , *et al.* Cooperative effects of *INK4a* and *ras* in melanoma susceptibility *in vivo* . Genes Dev 1997; 11: 2822–2834. 9353252 10.1101/gad.11.21.2822 PMC316663

[23] Eskandarpour M , Hashemi J , Kanter L , *et al.* Frequency of UV‐inducible NRAS mutations in melanomas of patients with germline CDKN2A mutations. J Natl Cancer Inst 2003; 95: 790–798. 12783933 10.1093/jnci/95.11.790

[24] Della Porta G. Cellular and molecular biology of melanoma. Semin Surg Oncol 1992; 8: 353–357. 1439444 10.1002/ssu.2980080604

[25] van't Veer LJ , Burgering BM , Versteeg R , *et al.* N‐ras mutations in human cutaneous melanoma from sun‐exposed body sites. Mol Cell Biol 1989; 9: 3114–3116. 2674680 10.1128/mcb.9.7.3114-3116.1989 PMC362784

[26] Davies H , Bignell GR , Cox C , *et al.* Mutations of the *BRAF* gene in human cancer. Nature 2002; 417: 949–954. 12068308 10.1038/nature00766

[27] Dossett LA , Kudchadkar RR , Zager JS . BRAF and MEK inhibition in melanoma. Expert Opin Drug Safety 2015; 14: 559–570. 10.1517/14740338.2015.1011618 25648338

[28] Krauthammer M , Kong Y , Ha BH , *et al.* Exome sequencing identifies recurrent somatic RAC1 mutations in melanoma. Nature Genet 2012; 44: 1006–1014. 22842228 10.1038/ng.2359 PMC3432702

[29] Cancer Genome Atlas Network . Genomic classification of cutaneous melanoma. Cell 2015; 161: 1681–1696. 26091043 10.1016/j.cell.2015.05.044 PMC4580370

[30] Hodis E , Watson IR , Kryukov GV , *et al.* A landscape of driver mutations in melanoma. Cell 2012; 150: 251–263. 22817889 10.1016/j.cell.2012.06.024 PMC3600117

[31] Wilsker D , Probst L , Wain HM , *et al.* Nomenclature of the ARID family of DNA‐binding proteins. Genomics 2005; 86: 242–251. 15922553 10.1016/j.ygeno.2005.03.013

[32] Hammond D , Zeng K , Espert A , et al. Melanoma‐associated mutations in protein phosphatase 6 cause chromosome instability and DNA damage owing to dysregulated Aurora‐A. J Cell Sci 2013; 126: 3429–3440. 23729733 10.1242/jcs.128397

[33] Fecher LA , Amaravadi RK , Flaherty KT . The MAPK pathway in melanoma. Curr Opin Oncol 2008; 20: 183–189. 18300768 10.1097/CCO.0b013e3282f5271c

[34] Turajlic S , Furney SJ , Lambros MB , et al. Whole genome sequencing of matched primary and metastatic acral melanomas. Genome Res 2012; 22: 196–207. 22183965 10.1101/gr.125591.111 PMC3266028

[35] Furney SJ , Turajlic S , Fenwick K , et al. Genomic characterisation of acral melanoma cell lines. Pigment Cell Melanoma Res 2012; 25: 488–492. 22578220 10.1111/j.1755-148X.2012.01016.x

[36] Pervaiz S , Cao J , Chao OS , *et al.* Activation of the RacGTPase inhibits apoptosis in human tumor cells. Oncogene 2001; 20: 6263–6268. 11593437 10.1038/sj.onc.1204840

[37] Fidler IJ . Selection of successive tumour lines for metastasis. Nature New Biol 1973; 242: 148–149. 4512654 10.1038/newbio242148a0

[38] Maslow DE . Tabulation of results on the heterogeneity of cellular characteristics among cells from B16 mouse melanoma cell lines with different colonization potentials. A summary of sixty reports. Invasion Metastasis 1989; 9: 182–191. 2656569

[39] Pawlowski A , Lea PJ . Human melanoma xenografts. Carcinog Compr Surv 1989; 11: 103–132. 2646013

[40] Kerbel RS . Human tumor xenografts as predictive preclinical models for anticancer drug activity in humans: better than commonly perceived – but they can be improved. Cancer Biol Ther 2003; 2: S134–S139. 14508091

[41] Khaled WT , Liu P . Cancer mouse models: past, present and future. Semin Cell Dev Biol 2014; 27: 54–60. 24718321 10.1016/j.semcdb.2014.04.003

[42] Quintana E , Piskounova E , Shackleton M , et al. Human melanoma metastasis in NSG mice correlates with clinical outcome in patients. Sci Transl Med 2012; 4:159ra149. 10.1126/scitranslmed.3004599 PMC4501487 23136044

[43] Einarsdottir BO , Bagge RO , Bhadury J , et al. Melanoma patient‐derived xenografts accurately model the disease and develop fast enough to guide treatment decisions. Oncotarget 2014; 5: 9609–9618. 25228592 10.18632/oncotarget.2445 PMC4259423

[44] Tanaka S , Saito Y , Kunisawa J , et al. Development of mature and functional human myeloid subsets in hematopoietic stem cell‐engrafted NOD/SCID/IL2rγKO mice. J Immunol 2012; 188: 6145–6155. 22611244 10.4049/jimmunol.1103660 PMC3370073

[45] Sharpless NE , Bardeesy N , Lee KH , et al. Loss of p16^Ink4a^ with retention of p19^Arf^ predisposes mice to tumorigenesis. Nature 2001; 413: 86–91. 11544531 10.1038/35092592

[46] Campagne C , Reyes‐Gomez E , Battistella M , et al. Histopathological atlas and proposed classification for melanocytic lesions in Tyr::NRas(Q61K); Cdkn2a(−/−) transgenic mice. Pigment Cell Melanoma Res 2013; 26: 735–742. 23647911 10.1111/pcmr.12115

[47] Dhomen N , Reis‐Filho JS , da Rocha Dias S , et al. Oncogenic Braf induces melanocyte senescence and melanoma in mice. Cancer Cell 2009; 15: 294–303. 19345328 10.1016/j.ccr.2009.02.022

[48] Perna D , Karreth FA , Rust AG , et al. BRAF inhibitor resistance mediated by the AKT pathway in an oncogenic BRAF mouse melanoma model. Proc Natl Acad Sci U S A 2015; 112: E536–E545. 25624498 10.1073/pnas.1418163112 PMC4330752

[49] Dankort D , Curley DP , Cartlidge RA , et al. *Braf* ^V600E^ cooperates with *Pten* loss to induce metastatic melanoma. Nature Genet 2009; 41: 544–552. 19282848 10.1038/ng.356 PMC2705918

[50] Noonan FP , Recio JA , Takayama H , et al. Neonatal sunburn and melanoma in mice. Nature 2001; 413: 271–272. 11565020 10.1038/35095108

[51] Gaffal E , Landsberg J , Bald T , *et al.* Neonatal UVB exposure accelerates melanoma growth and enhances distant metastases in Hgf‐Cdk4(R24C) C57BL/6 mice. Int J Cancer 2011; 129: 285–294. 21207411 10.1002/ijc.25913

[52] Bald T , Quast T , Landsberg J , et al. Ultraviolet‐radiation‐induced inflammation promotes angiotropism and metastasis in melanoma. Nature 2014; 507: 109–113. 24572365 10.1038/nature13111

[53] Jarrett SG , Novak M , Harris N , *et al.* NM23 deficiency promotes metastasis in a UV radiation‐induced mouse model of human melanoma. Clin Exp Metastasis 2013; 30: 25–36. 22699362 10.1007/s10585-012-9495-z PMC3547246

[54] Ha L , Ichikawa T , Anver M , et al. ARF functions as a melanoma tumor suppressor by inducing p53‐independent senescence. Proc Natl Acad Sci U S A 2007; 104: 10968–10973. 17576930 10.1073/pnas.0611638104 PMC1904138

[55] Moulton J (ed). Tumors in Domestic Animals (3rd edn). University of California Press: Berkeley and Los Angeles, 1990.

[56] Gillard M , Cadieu E , De Brito C , et al. Naturally occurring melanomas in dogs as models for non‐UV pathways of human melanomas. Pigment Cell Melanoma Res 2014; 27: 90–102. 24112648 10.1111/pcmr.12170

[57] Withrow SJ, Vail DM, Page RL (eds). Withrow and MacEwen's Small Animal Clinical Oncology (5th edn). Elsevier Saunders: St Louis, 2013.

[58] Suckow MA . Cancer vaccines: harnessing the potential of anti‐tumor immunity. Vet J 2013; 198: 28–33. 23850019 10.1016/j.tvjl.2013.06.005

[59] Grosenbaugh DA , Leard AT , Bergman PJ , et al. Safety and efficacy of a xenogeneic DNA vaccine encoding for human tyrosinase as adjunctive treatment for oral malignant melanoma in dogs following surgical excision of the primary tumor. Am J Vet Res 2011; 72: 1631–1638. 22126691 10.2460/ajvr.72.12.1631

[60] Vail DM . Levels of evidence in canine oncology trials – a case in point. Vet Comp Oncol 2013; 11: 167–168. 23909995 10.1111/vco.12058

[61] Ottnod JM , Smedley RC , Walshaw R , *et al.* A retrospective analysis of the efficacy of Oncept vaccine for the adjunct treatment of canine oral malignant melanoma. Vet Comp Oncol 2013; 11: 219–229. 23909996 10.1111/vco.12057

[62] Finocchiaro LME , Fondello C , Gil‐Cardeza ML , et al. Cytokine‐enhanced vaccine and interferon‐β plus suicide gene therapy as surgery adjuvant treatments for spontaneous canine melanoma. Hum Gene Ther 2015; 26: 367–376. 25762364 10.1089/hum.2014.130 PMC4492668

[63] Riccardo F , Iussich S , Maniscalco L , et al. CSPG4‐specific immunity and survival prolongation in dogs with oral malignant melanoma immunized with human CSPG4 DNA. Clin Cancer Res 2014; 20: 3753–3762. 24874834 10.1158/1078-0432.CCR-13-3042 PMC8656093

[64] London CA . Tyrosine kinase inhibitors in veterinary medicine. Top Companion Anim Med 2009; 24: 106–112. 19732728 10.1053/j.tcam.2009.02.002

[65] Lindblad‐Toh K , Wade CM , Mikkelsen TS , et al. Genome sequence, comparative analysis and haplotype structure of the domestic dog. Nature 2005; 438: 803–819. 16341006 10.1038/nature04338

[66] Mochizuki H , Kennedy K , Shapiro SG , *et al.* BRAF mutations in canine cancers. PloS One 2015; 10:e0129534. 10.1371/journal.pone.0129534 PMC4460039 26053201

[67] Poorman K , Borst L , Moroff S , et al. Comparative cytogenetic characterization of primary canine melanocytic lesions using array CGH and fluorescence *in situ* hybridization. Chromosome Res 2015; 23: 171–186. 25511566 10.1007/s10577-014-9444-6 PMC5462112

[68] Hutchinson L , Kirk R . High drug attrition rates – where are we going wrong? Nature Rev Clin Oncol 2011; 8: 189–190. 21448176 10.1038/nrclinonc.2011.34

[69] Khanna C , Lindblad‐Toh K , Vail D , et al. The dog as a cancer model. Nature Biotechnol 2006; 24: 1065–1066. 16964204 10.1038/nbt0906-1065b

[70] Gordon I , Paoloni M , Mazcko C , *et al.* The Comparative Oncology Trials Consortium: using spontaneously occurring cancers in dogs to inform the cancer drug development pathway. PLoS Med 2009; 6:e1000161. 10.1371/journal.pmed.1000161 PMC2753665 19823573

[71] Khanna C , London C , Vail D , *et al.* Guiding the optimal translation of new cancer treatments from canine to human cancer patients. Clin Cancer Res 2009; 15: 5671–5677. 19737961 10.1158/1078-0432.CCR-09-0719 PMC2748812

[72] Paoloni M , Khanna C . Translation of new cancer treatments from pet dogs to humans. Nature Rev Cancer 2008; 8: 147–156. 18202698 10.1038/nrc2273

[73] Simpson RM , Bastian BC , Michael HT , et al. Sporadic naturally occurring melanoma in dogs as a preclinical model for human melanoma. Pigment Cell Melanoma Res 2014; 27: 37–47. 24128326 10.1111/pcmr.12185 PMC4066658

[74] Knowles EJ , Tremaine WH , Pearson GR , *et al.* A database survey of equine tumours in the United Kingdom. Equine Vet J 2015; DOI: 10.1111/evj.12421. 10.1111/evj.12421 25594351

[75] Johnson PJ . Dermatologic tumors (excluding sarcoids). Vet Clin North Am Equine Pract 1998; 14: 625–658, viii. 9891728 10.1016/s0749-0739(17)30190-6

[76] Valentine BA . Survey of equine cutaneous neoplasia in the Pacific Northwest. J Vet Diagn Invest 2006; 18: 123–126. 16566271 10.1177/104063870601800121

[77] Smith SH , Goldschmidt MH , McManus PM . A comparative review of melanocytic neoplasms. Vet Pathol 2002; 39: 651–678. 12450197 10.1354/vp.39-6-651

[78] Valentine BA . Equine melanocytic tumors: a retrospective study of 53 horses (1988 to 1991). J Vet Intern Med 1995; 9: 291–297. 8531173 10.1111/j.1939-1676.1995.tb01087.x

[79] Schöniger S , Summers BA . Equine skin tumours in 20 horses resembling three variants of human melanocytic naevi. Vet Dermatol 2009; 20: 165–173. 19374725 10.1111/j.1365-3164.2009.00741.x

[80] MacFadyean J. Equine melanomatosis. J Comp Pathol Ther 1933; 46: 186–204.

[81] Knottenbelt DC , Patterson‐Kane JC , Snalune KL. Clinical Equine Oncology Elsevier: Amsterdam, 2015.

[82] MacGillivray KC , Sweeney RW , Del Piero F . Metastatic melanoma in horses. J Vet Intern Med 2002; 16: 452–456. 12141308 10.1892/0891-6640(2002)016<0452:mmih>2.3.co;2

[83] Campagne C , Julé S , Bernex F , et al. RACK1, a clue to the diagnosis of cutaneous melanomas in horses. BMC Vet Res 2012; 8: 95. 22747534 10.1186/1746-6148-8-95 PMC3543212

[84] Barnett KC , Platt H . Intraocular melanomata in the horse. Equine Vet J Suppl 1990; 22 **:** 76–82. 9079124 10.1111/j.2042-3306.1990.tb04718.x

[85] Dixon PM , Head KW . Equine nasal and paranasal sinus tumours: part 2: a contribution of 28 case reports. Vet J 1999; 157: 279–294. 10328839 10.1053/tvjl.1999.0371

[86] Head KW , Dixon PM . Equine nasal and paranasal sinus tumours. Part 1: review of the literature and tumour classification. Vet J 1999; 157: 261–278. 10328838 10.1053/tvjl.1998.0370

[87] Jiang L , Campagne C , Sundström E , et al. Constitutive activation of the ERK pathway in melanoma and skin melanocytes in Grey horses. BMC Cancer 2014; 14: 857. 25413220 10.1186/1471-2407-14-857 PMC4254013

[88] Sundström E , Komisarczuk AZ , Jiang L , et al. Identification of a melanocyte‐specific, microphthalmia‐associated transcription factor‐dependent regulatory element in the intronic duplication causing hair greying and melanoma in horses. Pigment Cell Melanoma Res 2012; 25: 28–36. 21883983 10.1111/j.1755-148X.2011.00902.x

[89] Teixeira RBC , Rendahl AK , Anderson SM , et al. Coat color genotypes and risk and severity of melanoma in gray quarter horses. J Vet Intern Med 2013; 27: 1201–1208. 23875712 10.1111/jvim.12133

[90] Seltenhammer MH , Sundström E , Meisslitzer‐Ruppitsch C , et al. Establishment and characterization of a primary and a metastatic melanoma cell line from Grey horses. In Vitro Cell Dev Biol Anim 2014; 50: 56–65. 23982913 10.1007/s11626-013-9678-1

[91] Zembowicz A , Carney JA , Mihm MC . Pigmented epithelioid melanocytoma: a low‐grade melanocytic tumor with metastatic potential indistinguishable from animal‐type melanoma and epithelioid blue nevus. Am J Surg Pathol 2004; 28: 31–40. 14707861 10.1097/00000478-200401000-00002

[92] Ludgate MW , Fullen DR , Lee J , et al. Animal‐type melanoma: a clinical and histopathological study of 22 cases from a single institution. Br J Dermatol 2010; 162: 129–136. 19709103 10.1111/j.1365-2133.2009.09271.x

[93] Zhao ZZ , Duffy DL , Thomas SA , *et al.* Polymorphisms in the syntaxin 17 gene are not associated with human cutaneous malignant melanoma. Melanoma Res 2009; 19: 80–86. 19209086 10.1097/CMR.0b013e328322fc45 PMC3665505

[94] Howe K , Clark MD , Torroja CF , et al. The zebrafish reference genome sequence and its relationship to the human genome. Nature 2013; 496: 498–503. 23594743 10.1038/nature12111 PMC3703927

[95] Ablain J , Durand EM , Yang S , *et al.* A CRISPR/Cas9 vector system for tissue‐specific gene disruption in zebrafish. Dev Cell 2015; 32: 756–764. 25752963 10.1016/j.devcel.2015.01.032 PMC4379706

[96] Irion U , Krauss J , Nüsslein‐Volhard C . Precise and efficient genome editing in zebrafish using the CRISPR/Cas9 system. Development 2014; 141: 4827–4830. 25411213 10.1242/dev.115584 PMC4299274

[97] Jao L‐E , Wente SR , Chen W . Efficient multiplex biallelic zebrafish genome editing using a CRISPR nuclease system. Proc Natl Acad Sci U S A 2013; 110: 13904–13909. 23918387 10.1073/pnas.1308335110 PMC3752207

[98] Chang N , Sun C , Gao L , et al. Genome editing with RNA‐guided Cas9 nuclease in zebrafish embryos. Cell Res 2013; 23: 465–472. 23528705 10.1038/cr.2013.45 PMC3616424

[99] Hwang WY , Fu Y , Reyon D , et al. Efficient genome editing in zebrafish using a CRISPR–Cas system. Nature Biotechnol 2013; 31: 227–229. 23360964 10.1038/nbt.2501 PMC3686313

[100] Patton EE , Widlund HR , Kutok JL , et al. BRAF mutations are sufficient to promote nevi formation and cooperate with p53 in the genesis of melanoma. Curr Biol 2005; 15: 249–254. 15694309 10.1016/j.cub.2005.01.031

[101] Ceol CJ , Houvras Y , Jane‐Valbuena J , et al. The histone methyltransferase SETDB1 is recurrently amplified in melanoma and accelerates its onset. Nature 2011; 471: 513–517. 21430779 10.1038/nature09806 PMC3348545

[102] White RM , Zon LI . Melanocytes in development, regeneration, and cancer. Cell Stem Cell 2008; 3: 242–252. 18786412 10.1016/j.stem.2008.08.005

[103] Zeng Z , Johnson SL , Lister JA , *et al.* Temperature‐sensitive splicing of *mitfa* by an intron mutation in zebrafish. Pigment Cell Melanoma Res 2015; 28: 229–232. 25469769 10.1111/pcmr.12336 PMC4333074

[104] Lister JA , Capper A , Zeng Z , et al. A conditional zebrafish MITF mutation reveals MITF levels are critical for melanoma promotion vs. regression *in vivo* . J Invest Dermatol 2014; 134: 133–140. 23831555 10.1038/jid.2013.293 PMC3898314

[105] Taylor KL , Lister JA , Zeng Z , et al. Differentiated melanocyte cell division occurs *in vivo* and is promoted by mutations in Mitf. Development 2011; 138: 3579–3589. 21771814 10.1242/dev.064014 PMC3143570

[106] Michailidou C , Jones M , Walker P , *et al.* Dissecting the roles of Raf‐ and PI3K‐signalling pathways in melanoma formation and progression in a zebrafish model. Dis Model Mech 2009; 2: 399–411. 19470611 10.1242/dmm.001149

[107] Dalton LE , Kamarashev J , Barinaga‐Rementeria Ramirez I , *et al.* Constitutive RAC activation is not sufficient to initiate melanocyte neoplasia but accelerates malignant progression. J Invest Dermatol 2013; 133: 1572–1581. 23337888 10.1038/jid.2013.23

[108] Santoriello C , Gennaro E , Anelli V , et al. Kita driven expression of oncogenic HRAS leads to early onset and highly penetrant melanoma in zebrafish. PloS One 2010; 5:e15170. 10.1371/journal.pone.0015170 PMC3000817 21170325

[109] Dovey M , White RM , Zon LI . Oncogenic NRAS cooperates with *p53* loss to generate melanoma in zebrafish. Zebrafish 2009; 6: 397–404. 19954345 10.1089/zeb.2009.0606 PMC2943216

[110] Yen J , White RM , Wedge DC , et al. The genetic heterogeneity and mutational burden of engineered melanomas in zebrafish models. Genome Biol 2013; 14: R113. 24148783 10.1186/gb-2013-14-10-r113 PMC3983654

[111] Feng Y , Renshaw S , Martin P . Live imaging of tumor initiation in zebrafish larvae reveals a trophic role for leukocyte‐derived PGE_2_ . Curr Biol 2012; 22: 1253–1259. 22658594 10.1016/j.cub.2012.05.010 PMC3398414

[112] Feng Y , Santoriello C , Mione M , *et al.* Live imaging of innate immune cell sensing of transformed cells in zebrafish larvae: parallels between tumor initiation and wound inflammation. PLoS Biol 2010; 8:e1000562. 10.1371/journal.pbio.1000562 PMC3001901 21179501

[113] Taylor AM , Zon LI . Zebrafish tumor assays: the state of transplantation. Zebrafish 2009; 6: 339–346. 20047467 10.1089/zeb.2009.0607 PMC2809423

[114] Díez‐Torre A , Andrade R , Eguizábal C , et al. Reprogramming of melanoma cells by embryonic microenvironments. Int J Dev Biol 2009; 53: 1563–1568. 19924629 10.1387/ijdb.093021ad

[115] Haldi M , Ton C , Seng WL , *et al.* Human melanoma cells transplanted into zebrafish proliferate, migrate, produce melanin, form masses and stimulate angiogenesis in zebrafish. Angiogenesis 2006; 9: 139–151. 17051341 10.1007/s10456-006-9040-2

[116] Topczewska JM , Postovit L‐M , Margaryan NV , et al. Embryonic and tumorigenic pathways converge via Nodal signaling: role in melanoma aggressiveness. Nature Med 2006; 12: 925–932. 16892036 10.1038/nm1448

[117] Lee LMJ , Seftor EA , Bonde G , *et al.* The fate of human malignant melanoma cells transplanted into zebrafish embryos: assessment of migration and cell division in the absence of tumor formation. Dev Dyn 2005; 233: 1560–1570. 15968639 10.1002/dvdy.20471

[118] Lawson ND , Weinstein BM . *In vivo* imaging of embryonic vascular development using transgenic zebrafish. Dev Biol 2002; 248: 307–318. 12167406 10.1006/dbio.2002.0711

[119] Hoffman SJ , Psaltis PJ , Clark KJ , et al. An *in vivo* method to quantify lymphangiogenesis in zebrafish. PloS One 2012; 7:e45240. 10.1371/journal.pone.0045240 PMC3441694 23028871

[120] Chapman A , Fernandez del Ama L , Ferguson J , *et al.* Heterogeneous tumor subpopulations cooperate to drive invasion. Cell Rep 2014; 8: 688–695. 25066122 10.1016/j.celrep.2014.06.045 PMC4542310

[121] Spaink HP , Cui C , Wiweger MI , et al. Robotic injection of zebrafish embryos for high‐throughput screening in disease models. Methods 2013; 62: 246–254. 23769806 10.1016/j.ymeth.2013.06.002

[122] He S , Lamers GE , Beenakker J‐WM , et al. Neutrophil‐mediated experimental metastasis is enhanced by VEGFR inhibition in a zebrafish xenograft model. J Pathol 2012; 227: 431–445. 22374800 10.1002/path.4013 PMC3504093

[123] Li P , White RM , Zon LI . Transplantation in zebrafish. Methods Cell Biol 2011; 105: 403–417. 21951540 10.1016/B978-0-12-381320-6.00017-5

[124] White RM , Sessa A , Burke C , et al. Transparent adult zebrafish as a tool for *in vivo* transplantation analysis. Cell Stem Cell 2008; 2: 183–189. 18371439 10.1016/j.stem.2007.11.002 PMC2292119

[125] Rennekamp AJ , Peterson RT . 15 years of zebrafish chemical screening. Curr Opin Chem Biol 2015; 24: 58–70. 25461724 10.1016/j.cbpa.2014.10.025 PMC4339096

[126] White RM , Cech J , Ratanasirintrawoot S , et al. DHODH modulates transcriptional elongation in the neural crest and melanoma. Nature 2011; 471: 518–522. 21430780 10.1038/nature09882 PMC3759979

[127] Zhou L , Ishizaki H , Spitzer M , et al. ALDH2 mediates 5‐nitrofuran activity in multiple species. Chem Biol 2012; 19: 883–892. 22840776 10.1016/j.chembiol.2012.05.017 PMC3684953

[128] White R , Rose K , Zon L . Zebrafish cancer: the state of the art and the path forward. Nature Rev Cancer 2013; 13: 624–636. 23969693 10.1038/nrc3589 PMC6040891

[129] Zon L. Translational research: the path for bringing discovery to patients. Cell Stem Cell 2014; 14: 146–148. 24506882 10.1016/j.stem.2014.01.004 PMC5972520

[130] Millikan LE , Boylon JL , Hook RR , *et al.* Melanoma in Sinclair swine: a new animal model. J Invest Dermatol 1974; 62: 20–30. 4809019 10.1111/1523-1747.ep12676714

[131] Vincent‐Naulleau S , Le Chalony C , Leplat J‐J , et al. Clinical and histopathological characterization of cutaneous melanomas in the melanoblastoma‐bearing Libechov minipig model. Pigment Cell Res 2004; 17: 24–35. 14717842 10.1046/j.1600-0749.2003.00101.x

[132] Chudnovsky Y , Adams AE , Robbins PB , *et al.* Use of human tissue to assess the oncogenic activity of melanoma‐associated mutations. Nature Genet 2005; 37: 745–749. 15951821 10.1038/ng1586 PMC3063773

[133] Patton EE , Mitchell DL , Nairn RS . Genetic and environmental melanoma models in fish. Pigment Cell Melanoma Res 2010; 23: 314–337. 20230482 10.1111/j.1755-148X.2010.00693.x PMC2881310

[134] Harrington M. Marsupials that model melanoma. Lab Anim 2015; 44: 53. 10.1038/laban.698 25602390

[135] Ackermann J , Frutschi M , Kaloulis K , *et al.* Metastasizing melanoma formation caused by expression of activated N‐RasQ61K on an INK4a‐deficient background. Cancer Res 2005; 65: 4005–4011. 15899789 10.1158/0008-5472.CAN-04-2970

[136] Iwamoto T , Takahashi M , Ito M , et al. Aberrant melanogenesis and melanocytic tumour development in transgenic mice that carry a metallothionein/ret fusion gene. EMBO J 1991; 10: 3167–3175. 1915289 10.1002/j.1460-2075.1991.tb04878.x PMC453039

[137] Kato M , Takahashi M , Akhand AA , et al. Transgenic mouse model for skin malignant melanoma. Oncogene 1998; 17: 1885–1888. 9778055 10.1038/sj.onc.1202077

[138] Kumasaka MY , Yajima I , Hossain K , et al. A novel mouse model for *de novo* melanoma. Cancer Res 2010; 70: 24–29. 20048069 10.1158/0008-5472.CAN-09-2838

[139] Takayama H , LaRochelle WJ , Sharp R , et al. Diverse tumorigenesis associated with aberrant development in mice overexpressing hepatocyte growth factor/scatter factor. Proc Natl Acad Sci U S A 1997; 94: 701–706. 9012848 10.1073/pnas.94.2.701 PMC19577

[140] Noonan FP , Recio JA , Takayama H , et al. Neonatal sunburn and melanoma in mice. Nature 2001; 413: 271–272. 11565020 10.1038/35095108

[141] Tormo D , Ferrer A , Bosch P , et al. Therapeutic efficacy of antigen‐specific vaccination and toll‐like receptor stimulation against established transplanted and autochthonous melanoma in mice. Cancer Res 2006; 66: 5427–5435. 16707471 10.1158/0008-5472.CAN-06-0399

[142] Vidwans SJ , Flaherty KT , Fisher DE , *et al.* A melanoma molecular disease model. PloS One 2011; 6 **:** e18257. 10.1371/journal.pone.0018257 PMC3068163 21479172

